# The Universal 3D QSAR Model for Dopamine D_2_ Receptor Antagonists

**DOI:** 10.3390/ijms20184555

**Published:** 2019-09-14

**Authors:** Agata Zięba, Justyna Żuk, Damian Bartuzi, Dariusz Matosiuk, Antti Poso, Agnieszka A. Kaczor

**Affiliations:** 1 Department of Synthesis and Chemical Technology of Pharmaceutical Substances with Computer Modeling Laboratory, Faculty of Pharmacy with Division of Medical Analytics, 4A Chodzki St, PL-20059 Lublin, Poland; 2 School of Pharmacy, University of Eastern Finland, Yliopistonranta 1, P.O. Box 1627, FI-70211 Kuopio, Finland; 3 Department. of Internal Medicine VIII, University Hospital Tübingen, Otfried-Müller-Strasse 14, 72076 Tübingen, Germany

**Keywords:** 3D-QSAR, CoMFA model, dopamine D_2_ receptor, dopamine D_2_ receptor antagonists

## Abstract

In order to search for novel antipsychotics acting through the D_2_ receptor, it is necessary to know the structure–activity relationships for dopamine D_2_ receptor antagonists. In this context, we constructed the universal three-dimensional quantitative structure–activity relationship (3D- QSAR) model for competitive dopamine D_2_ receptor antagonists. We took 176 compounds from chemically different groups characterized by the half maximal inhibitory concentration (IC_50_)from the CHEMBL database and docked them to the X-ray structure of the human D_2_ receptor in the inactive state. Selected docking poses were applied for Comparative Molecular Field Analysis (CoMFA) alignment. The obtained CoMFA model is characterized by a cross-validated coefficient Q^2^ of 0.76 with an optimal component of 5, R^2^ of 0.92, and an F value of 338.9. The steric and electrostatic field contributions are 67.4% and 32.6%, respectively. The statistics obtained prove that the CoMFA model is significant. Next, the IC_50_ of the 16 compounds from the test set was predicted with R^2^ of 0.95. Finally, a progressive scrambling test was carried out for additional validation. The CoMFA fields were mapped onto the dopamine D_2_ receptor binding site, which enabled a discussion of the structure–activity relationship based on ligand–receptor interactions. In particular, it was found that one of the desired steric interactions covers the area of a putative common allosteric pocket suggested for some other G protein-coupled receptors (GPCRs), which would suggest that some of the known dopamine receptor antagonists are bitopic in their essence. The CoMFA model can be applied to predict the potential activity of novel dopamine D_2_ receptor antagonists.

## 1. Introduction

The dopamine D_2_ receptor is the main molecular target of all antipsychotics currently available on the pharmaceutical market. In particular, the first- (typical) and second- generation (atypical) neuroleptics are dopamine D_2_ receptor antagonists, whereas the third-generation drugs are partial or biased agonists of this receptor [1]. Although attempts have been made to search for drugs against schizophrenia beyond the dopaminergic hypothesis of this disease, none of the investigated compounds were completed successfully in the clinical studies [2]. Thus, in order to search for novel antipsychotics, it is necessary to investigate structure–activity relationships for dopamine D_2_ receptor ligands. The available X-ray structure of the human dopamine D_2_ receptor in its inactive state in complex with risperidone (PDB ID: 6CM4) [3] also enables an investigation of drug–receptor interactions at the molecular level.

Three-dimensional quantitative structure–activity relationship (3D-QSAR) methods are crucial for drug discovery and ligand-based molecular modeling. These techniques are particularly useful if the 3D structure of a molecular target is not available. 3D-QSAR techniques are applied to determine the relationship between the molecular properties and observed pharmacological activities of a group of congeneric compounds [4,5]. A widely used 3D-QSAR approach is Comparative Molecular Field Analysis (CoMFA), which uses statistical methods to correlate steric and electrostatic properties of a series of molecules with their pharmacological activities.

Taking advantage of available data on the dopamine D_2_ receptor and its ligands, we decided to construct a universal 3D-QSAR model for D_2_ receptor antagonists from different chemical groups using molecular docking-based alignment. It has been reported that the quality of molecular alignment is a key factor in determining the quality of the resulting CoMFA model [6]. Ligand-based alignment, following the pharmacophore theory that similar molecules share similar orientation in the binding site, may lead in certain cases to incorrect models [4,7]. In this context, the best data sources for molecular alignment are the ligand–receptor X-ray structures, as demonstrated by Urniaz and Jozwiak for α-amino-3-hydroxy-5-methyl-4-isoxazolepropionic acid (AMPA) receptor ligands [4]. However, in the case of G protein-coupled receptors (GPCRs), the number of crystallized ligand–receptor complexes is limited. For GPCRs [8] and many other proteins [9], molecular docking remains the method of choice to obtain molecular alignment taking into consideration ligand–receptor interactions.

Our work constitutes a novel extension to previously published QSAR models for dopamine D_2_ receptor ligands [10,11]. Fatemi and Dorostkar [10] constructed the nonlinear and linear QSAR models on a series of 6-methoxybenzamides applying the artificial neural network (ANN) and multiple linear regressions (MLR), respectively. Wang et al. [11] built Comparative Molecular Similarity Indices Analysis (CoMSiA) models for dopamine D_2_/D_3_ receptor ligands utilizing alignment based on molecular docking to homology models of respective receptors [12]. However, they worked on a series of 163 compounds which all followed one structural pattern and could be described by one general formula, as shown in Table S1 in the Supplementary Information to this article. The advantage of our QSAR model is the usage of a few series of structurally unrelated compounds to obtain the model which can be termed universal. Moreover, one of the aims of our work was to map the obtained CoMFA fields onto the receptor 3D structure, which enabled us to draw interesting conclusions about the binding of dopamine D_2_ receptor antagonists.

## 2. Results and Discussion

### 2.1. Studied Compounds

The studied compounds were selected from the CHEMBL database [13] based on the availability of IC_50_ as an in vitro measure of their antagonistic activity (cAMP assay) towards the dopamine D_2_ receptor. Most compounds belong to the benzamide or arylpiperazine families. In total, 176 compounds were studied, and they were divided into a training set (160 compounds) and a test set (16 compounds), as shown in Table S1 in the Supplementary Information. The compounds were ordered and numbered according to their decreasing experimental pIC_50_ (negative of the base 10 logarithm of the half maximal inhibitory concentration) values.

### 2.2. Molecular Docking

Compounds **1–176**, as shown in Table S1 in the Supplementary Information, were docked to the orthosteric binding site of the human dopamine D_2_ receptor X-ray structure in the inactive state in complex with risperidone (PDB ID: 6CM4) [3]. The molecular docking was performed with the standard precision (SP) approach of Glide of Schrödinger software v. 2018-2 with default settings using the grid based on the cocrystallized ligand, risperidone, as previously reported [14].

**Figure 1:**
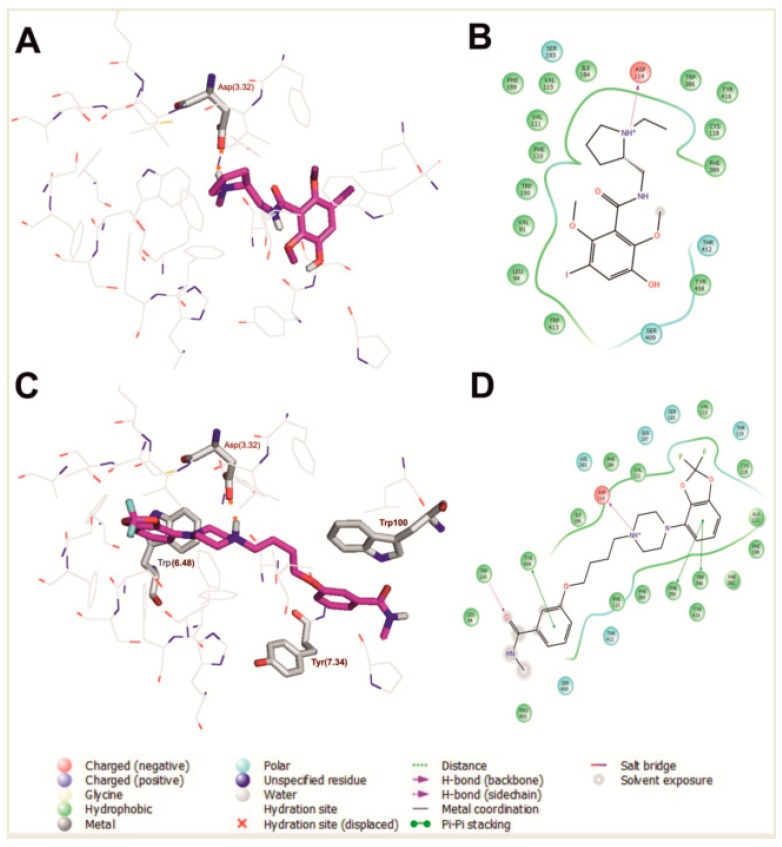
Compound **1** (**A**,**B**) and compound **17** (**C**,**D**) in the orthosteric binding site of the human dopamine D_2_ receptor (selected molecular docking poses). (**A**,**C**) 3D view of the binding pocket. Ligands are shown as sticks with magenta representing carbon atoms. Receptors are shown as wires with grey representing carbon atoms, while the main interacting residues are represented as sticks. Hydrogen bonds are depicted as red dashed lines. Nonpolar hydrogen atoms are not shown for clarity. (**B**,**D**) 2D view of the binding pocket.

The selected binding poses of the most active benzamide (**1**) and arylpiperazine (**17**) are shown in Figure 1. The main contact of risperidone, benzamide (**1**), and arylpiperazine (**17**) with the human dopamine D_2_ receptor is an electrostatic interaction between the protonatable nitrogen of the ligand and the conserved Asp 114 (3.32) (Ballesteros–Weinstein nomenclature) [15] from the third transmembrane helix, as is typical for orthosteric ligands of aminergic GPCRs. Moreover, in the case of compound (**17**), Trp 386 (6.48), Tyr 408 (7.34), and Trp 100 are also important for interaction with the receptor, as previously reported for a multi-target ligand of aminergic GPCRs, namely D2AAK1 [16,17] and D2AAK1 derivatives [14,18].

### 2.3. Molecular Alignment

The quality of molecular alignment is the key factor affecting the resulting 3D-QSAR model. It was demonstrated that the alignment based on X-ray structures of ligand–receptor complexes leads to the best QSAR statistics [4]. When crystallographic data is not available, molecular docking can be a data source of ligand–receptor complexes for molecular alignment [8,9]. Moreover, it can be a method to align compounds belonging to different structural groups.

In order to align compounds **1**–**176**, the docking poses of these compounds with the protonatable nitrogen of the ligand interacting with Asp 114 (3.32) of the receptor were selected. The binding poses of different compounds were chosen in order to align the protonatable nitrogen atom of all ligands and then other moieties of similar ligands, if possible.

### 2.4. CoMFA Statistics

**Figure 2:**
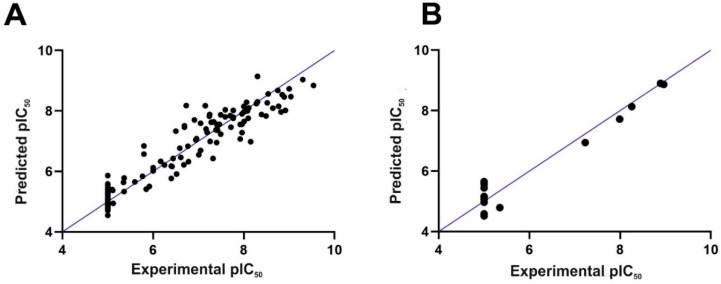
The experimental versus predicted pIC_50_ (negative of the base 10 logarithm of the half maximal inhibitory concentration) values for the training set (**A**) and test set (**B**).

The 3D-QSAR CoMFA model was constructed applying Sybyl-X v. 2.1. The following statistics were obtained for the CoMFA model: a cross-validated coefficient Q^2^ of 0.76 with an optimal component of 5, R^2^ of 0.92, and an F value of 338.9, which means that the model is statistically significant. In particular, the good internal predictability of the model is supported by the value of the cross-validated coefficient Q^2^ (above 0.5). The steric and electrostatic field contributions were 67.4% and 32.6%, respectively. The comparison of experimental and predicted IC_50_ values followed by the residual values are shown in Table S1 in the Supplementary Information. Importantly, the experimental and computed pIC_50_ values do not differ considerably from each other (in most cases, by no more than 1 logarithmic unit). Figure 2 presents the obtained correlation between the experimental and computed pIC_50_ values for the training set, as shown in Figure 2A, and the test set, as shown in Figure 2B.

### 2.5. Validation of CoMFA Model

The classical method of CoMFA model validation is activity prediction of the external test set of compounds. In our case, 16 compounds from the test were predicted, as shown in Table S1 and Figure 2B. The compounds from the training set were predicted with the R^2^ close to the R^2^ of the compounds from the test set (0.92 versus 0.95, respectively). Next, a progressive scrambling test was carried out as the next step of the CoMFA model validation (see reference [19] for details). In a stable model, the dQ^2^/dR^2^yy value should not be above 1.2 (ideally, it should be 1) [19]. This approach was used for the CoMFA model to check the number of components applied to construct the model and to investigate the cross-validation against the possibility of such redundancy occurring in the training set [19]. Table 1 lists the results of the progressive scrambling of the CoMFA model. Q^2^ values above 0.35 prove that the original, unperturbed model is robust [20]. The values of dQ^2^/dR^2^yy indicate that with up to five components (as in the constructed model), the model is stable.

### 2.6. Contour Map and Its Mapping onto Receptor Structure

**Figure 3:**
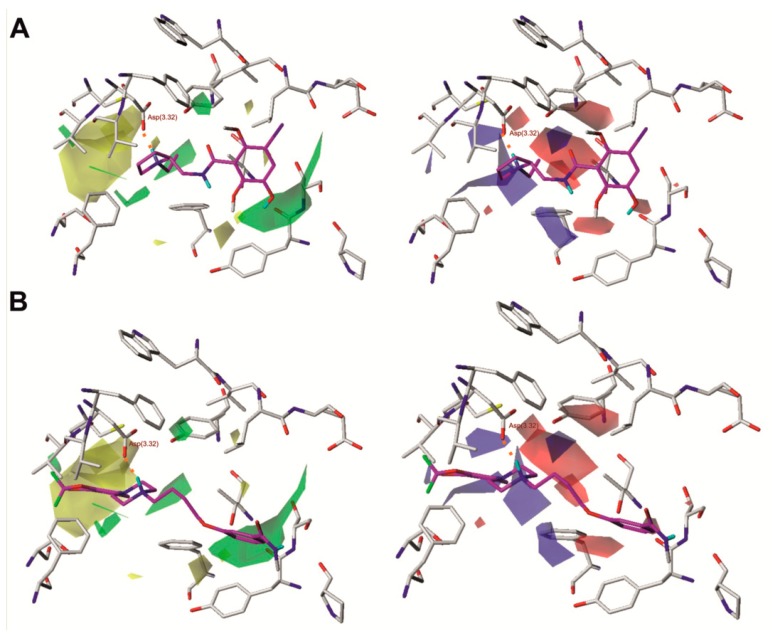
Comparative molecular field analysis (CoMFA) steric and electrostatic fields projected on the 3D structure of the dopamine D_2_ receptor in complex with compound **1** (**A**) and compound **17** (**B**). Ligands are shown as sticks with magenta representing carbon atoms. Receptors are shown as wires with grey representing carbon atoms, while the main interacting residues are represented as sticks. Hydrogen bonds are depicted as red dashed lines. Nonpolar hydrogen atoms are not shown for clarity.

Figure 3 shows the steric and electrostatic contour maps obtained via CoMFA modeling mapped onto the X-ray structure of dopamine D_2_ receptor. Steric contour maps supply information about the spatial volume of substituted groups in various arrangements. The most notable features are two large steric fields, one favourable and one undesired, located at the opposite sides of the ligand. Some beneficial steric interactions are also seen in the direct neighborhood of the protonated nitrogen atom of the ligands. Desired polar interactions in the area are expected and quite obvious, resulting from the salt bridge interaction anchoring the ligands in the aminergic receptors. However, there are also some additional polar interactions in the neighborhood of the amide moiety of benzamides.

Analysis of molecular fields superposed on the protein structure reveals some interesting relationships, as shown in Figure 3. Most apparent and intriguing is the large volume of unfavorable steric interactions in the most buried part of the binding pocket, which indicates that bulky substituents are generally not desired below the Asp(3.32) residue in the z-axis. Substituents located in this region of the binding site in other GPCRs were suggested to affect water exchange in a binding pocket, and the exchange intensity was considered as a factor discriminating between agonists and antagonists [21]. This exclusion volume favors pyrrolidine derivatives, which generally presented shallow docking poses and do not violate the undesired space. Moreover, 2-ethylpyrrolidine derivatives also fill the two desired steric fields at the neighborhood of Asp(3.32) with the ethyl moiety and the aliphatic fragment of the heterocyclic ring. However, there are also fields of desired steric interaction as well as desired negative charge around and below the abovementioned large undesired volume, and they are not accessible in any other way than by violating this space. This suggests that unfavorable influence of a deeply penetrating moiety can be diminished by its appropriate spatial arrangement, or that the unfavorable exclusion volume may stem from the choice of ligands, as a number of arylpiperazines with considerable D_2_ receptor affinity are known. In particular, the desired volumes are located at the opposite sides of the large undesired volume, which suggests that long and flat substituents like substituted aromatic rings are more acceptable. Unfortunately, detailed inspection of the docking positions from the training set does not provide a clear answer. The field indicating a desired negative charge is located more deeply, near the Ser(5.46) and Thr(3.37) residues, and shows that polar interaction with these spots improves inhibition. However, there is no field indicating that positive charge is desired in this area, which suggests that not any polar interactions should be engaged, but it also should induce the appropriate orientation of residues. It can be at least partially explained by the fact that only those properties with clear variation will be visible in any QSAR and especially in CoMFA.

Another large field, indicating favorable steric interactions of bulky molecules, is found near the top of transmembrane helices 1, 2 and 7 (TM1, TM2, and TM7). It is surrounded by some much smaller undesirable steric fields; however, those are typically created by small variations in alignments and as such are not very reliable. In some docking results, the favorable area is occupied when a large ligand is bound, and in such cases, it spans both the desirable steric field in the neighborhood of the extracellular loops (ECL) and the undesired field in the buried region of the binding pocket. However, there are also compounds, including **2**, **22**, and **33**, where only the extracellular desired field is partially or completely occupied by a ligand that remains relatively shallowly bound without violation of the buried undesired steric field. Interestingly, the area at the top of TM1, TM2, and TM7 is suggested to encompass an allosteric pocket in the opioid receptors [22,23]. In the light of these findings, if one assumes that the allosteric pocket could be conserved among a number of Class A GPCRs, this group of D_2_ antagonists could be considered bitopic ligands. Interestingly, in this region, there is also a desirable negative charge field which seems to be related to Ser(7.36) (7.36 × 35 in the GPCRdb numbering scheme; https://www.gpcrdb.org), which is also in a region that suggests it plays a role in allosteric signal transmission [22,23,24,25]. According to this molecular field analysis, such compounds are the most promising leads for more potent derivatives.

Notably, there is a desired positive charge field in the neighborhood of the His(6.55) residue, which suggests an interaction with hybridized sp^2^ electrons of heterocyclic nitrogen. The spatial arrangement of the molecular fields and protein structure suggest that such interaction is most probable when the τ nitrogen is not protonated. This leads to the conclusion that in the in silico studies on the D_2_ receptor, e.g., the virtual screening or molecular dynamics of antagonists, the histidine should be prepared in a π protonation state.

An interesting pattern of desired positive and desired negative charges can be found in the neighborhood of the conserved Trp(6.48), Phe(6.51), and Tyr(7.43), where appropriate charge distribution in a ligand seems to govern conformation of these residues via interactions with π electrons or regions of lower electron density at ring edges.

## 3. Materials and Methods

### 3.1. Selection and Preparation of Compounds

The chemical compounds for this study were taken from the CHEMBL database [13]. There were 176 dopamine D_2_ receptor antagonists from chemically different groups, mainly benzamides and arylpiperazines, characterized by IC_50_ which were selected. In the case of compounds with IC_50_ above 100,000 nM, when the measurement method could not register a precise IC_50_ value, the pIC_50_ was arbitrarily assumed as 5, similarly as was previously done for ABHD6 inhibitors [9]. This allowed the inclusion of inactive compounds in the dataset, and thus to extend the study. The compounds were prepared with the LigPrep [26] module from Schrödinger software v. 2018-2. In order to sample different protonation states of ligands at physiological pH, the Epik [27] module of Schrödinger software was used.

### 3.2. Molecular Docking

The compounds were docked with Glide [28] from Schrödinger software v. 2018-2 to the novel X-ray structure of the human dopamine D_2_ receptor in the inactive state (PDB ID: 6CM4) [3]. The grid was generated based on the co-crystallized ligand, risperidone, at default settings. The standard precision (SP) method of Glide molecular docking was applied. There were 20 poses generated for each compound.

### 3.3. CoMFA Studies

Molecular alignment for CoMFA studies was performed based on molecular docking results. For this purpose, only docking poses where a protonatable nitrogen atom of the ligand interacts with the conserved aspartate Asp114 (3.32) from the third transmembrane helix were considered. The binding poses were selected to enable superposition of chemically equivalent moieties from structurally different classes of compounds. The set of 176 compounds was divided into a training set (160 molecules) and a test set (16 molecules, 10% of the training set). The division of compounds between the training set and test set was performed to satisfy the following criteria: (i) activity of compounds in both training and test sets expressed as pIC_50_ spans 5 orders of magnitudes from 5 to over 9, as recommended for 3D-QSAR studies; (2) the structural diversity of the compounds is ensured in both sets.

The CoMFA model was constructed using the QSAR module in Sybyl-X v. 2.1. The standard Tripos force field was applied for CoMFA modeling with Gasteiger–Hückel point charges and the default sp^3^ carbon probe with point charge +1.0, as described previously [8,9]. The optimal number of components was designated so that cross-validated R^2^ (Q^2^) values were maximal and the standard error of prediction was minimal, as previously reported [8,9].

Partial least squares (PLS) analysis was used to correlate the CoMFA fields linearly to pIC_50_ activity values. A cross-validation analysis was carried out using the leave-one-out (LOO) method, in which one compound is removed from the data set and its activity is predicted applying the model derived from the remaining compounds, as reported previously [8,9]. The model characterized by the highest Q^2^, the optimum number of components (ONC), and the lowest standard error of prediction was taken for further analysis. In addition, the statistical significance of the model was described by the standard error of estimate (SEE) and the probability value (F value).

The predictive capability of the 3D-QSAR model was evaluated with the external test set of 16 compounds. Moreover, a progressive scrambling validation test was also carried out. The CoMFA contour maps were mapped onto the binding site of the dopamine D_2_ receptor and the structure–activity relationship was discussed in the context of ligand–receptor interactions.

The study was limited to the CoMFA model only, although the CoMSiA is also available in Sybyl-X. These methods are similar and both have their advantages and disadvantages. CoMSiA studies performed by Wang et al. [11] for a significantly less structurally differentiated set of dopamine D_2_ receptor ligands resulted in much worse statistical parameters.

## 4. Conclusions

The presented Comparative Molecular Field Analysis (CoMFA) constitutes the first universal QSAR model for dopamine D_2_ receptor antagonists constructed on molecular docking-based alignment. It is characterized with very high statistical significance (R^2^ = 0.92, Q^2^ = 0.76). Except for providing interesting insights into structural requirements for this class of ligands, it also resulted in some data unexpectedly supporting suggestions regarding the presence of a common allosteric site in GPCRs, which therefore revealed some more details on the mechanisms of some of the investigated orthosteric antagonists.

## Figures and Tables

**Table 1:** Progressive scrambling test results for the comparative molecular field analysis (CoMFA) model.

Components	Q^2^	cSDEP	dQ^2^/dR^2^yy
2	0.54	0.95	0.91
3	0.61	0.88	0.94
4	0.62	0.86	1.14
5	0.61	0.87	1.19
6	0.59	0.89	1.30
7	0.57	0.93	1.32

## Supplementary Materials

Supplementary materials can be found at https://www.mdpi.com/1422-0067/20/18/4555/s1.

## Abbreviations

AMPA	α-Amino-3-hydroxy-5-methyl-4-isoxazolepropionic acid
ANN	Artificial neural network
CoMFA	Comparative molecular field analysis
CoMSiA	Comparative molecular similarity indices analysis
ECL	Extracellular loop
LOO	Leave-one-out
MLR	Multiple linear regressions
GPCRs	G protein-coupled receptors
ONC	Optimum number of components
PLS	Partial least square
QSAR	Quantitative structure–activity relationship
SEE	Standard error of estimate
SP	Standard precision
TM	Transmembrane helix

## References

[1] Stępnicki P., Kondej M., Kaczor A.A. (2018). Current concepts and treatments of schizophrenia. Molecules.

[2] Yang A.C., Tsai S.-J. (2017). New targets for schizophrenia treatment beyond the dopamine hypothesis. Int. J. Mol. Sci..

[3] Wang S., Che T., Levit A., Shoichet B.K., Wacker D., Roth B.L. (2018). Structure of the D2 dopamine receptor bound to the atypical antipsychotic drug risperidone. Nature.

[4] Urniaż R.D., Jóźwiak K. (2013). X-ray crystallographic structures as a source of ligand alignment in 3D-QSAR. J. Chem. Inf. Model..

[5] Kubinyi H. (1995). Strategies and recent technologies in drug discovery. Pharmazie.

[6] Cramer R.D., Patterson D.E., Bunce J.D. (1988). Comparative molecular field analysis (CoMFA). 1. Effect of shape on binding of steroids to carrier proteins. J. Am. Chem. Soc..

[7] Clark R.D., Abrahamian E. (2009). Using a staged multi-objective optimization approach to find selective pharmacophore models. J. Comput. Aided Msol. Des..

[8] Kaczor A.A., Żuk J., Matosiuk D. (2018). Comparative molecular field analysis and molecular dynamics studies of the dopamine D2 receptor antagonists without a protonatable nitrogen atom. Med. Chem. Res..

[9] Kaczor A.A., Targowska-Duda K.M., Patel J.Z., Laitinen T., Parkkari T., Adams Y., Nevalainen T.J., Poso A. (2015). Comparative molecular field analysis and molecular dynamics studies of α/β hydrolase domain containing 6 (ABHD6) inhibitors. J. Mol. Model..

[10] Fatemi M.H., Dorostkar F. (2010). QSAR prediction of D2 receptor antagonistic activity of 6-methoxy benzamides. Eur. J. Med. Chem..

[11] Wang Q., Mach R.H., Luedtke R.R., Reichert D.E. (2010). Subtype selectivity of dopamine receptor ligands: Insights from structure and ligand-based methods. J. Chem. Inf. Model..

[12] Luedtke R.R., Mishra Y., Wang Q., Griffin S.A., Bell-Horner C., Taylor M., Vangveravong S., Dillon G.H., Huang R.Q., Reichert D.E. (2012). Comparison of the binding and functional properties of two structurally different D2 dopamine receptor subtype selective compounds. ACS Chem. Neurosci..

[13] Gaulton A., Hersey A., Nowotka M., Bento A.P., Chambers J., Mendez D., Mutowo P., Atkinson F., Bellis L.J., Cibrián-Uhalte E. (2017). The ChEMBL database in 2017. Nucleic Acids Res..

[14] Kondej M., Wróbel T.M., Silva A.G., Stępnicki P., Koszła O., Kędzierska E., Bartyzel A., Biała G., Matosiuk D., Loza M.I. (2019). Synthesis, pharmacological and structural studies of 5-substituted-3-(1-arylmethyl-1,2,3,6-tetrahydropyridin-4-yl)-1H-indoles as multi-target ligands of aminergic GPCRs. Eur. J. Med. Chem..

[15] Ballesteros J.A., Weinstein H. (1995). Integrated methods for the construction of three-dimensional models and computational probing of structure-function relations in G protein-coupled receptors. Methods Neurosci..

[16] Kaczor A.A., Silva A.G., Loza M.I., Kolb P., Castro M., Poso A. (2016). Structure-Based Virtual Screening for Dopamine D2 Receptor Ligands as Potential Antipsychotics. ChemMedChem.

[17] Kaczor A.A., Targowska-Duda K.M., Budzyńska B., Biała G., Silva A.G., Castro M. (2016). In vitro, molecular modeling and behavioral studies of 3-{[4-(5-methoxy-1H-indol-3-yl)-1,2,3,6-tetrahydropyridin-1-yl]methyl}-1,2-dihydroquinolin-2-one (D2AAK1) as a potential antipsychotic. Neurochem. Int..

[18] Kondej M., Bartyzel A., Pitucha M., Wróbel T.M., Silva A.G., Matosiuk D., Castro M., Kaczor A.A. (2018). Synthesis, Structural and Thermal Studies of 3-(1-Benzyl-1,2,3,6-tetrahydropyridin-4-yl)-5-ethoxy-1H-indole (D2AAK1_3) as Dopamine D₂ Receptor Ligand. Molecules.

[19] Hevener K.E., Ball D.M., Buolamwini J.K., Lee R.E. (2008). Quantitative structure-activity relationship studies on nitrofuranyl anti-tubercular agents. Bioorg. Med. Chem..

[20] Clark R.D., Fox P.C. (2004). Statistical variation in progressive scrambling. J. Comput. Aided Mol. Des..

[21] Yuan S., Vogel H., Filipek S. (2013). The role of water and sodium ions in the activation of the μ-opioid receptor. Angew. Chem..

[22] Bartuzi D., Kaczor A.A., Matosiuk D. (2016). Interplay between Two Allosteric Sites and Their Influence on Agonist Binding in Human μ Opioid Receptor. J. Chem. Inf. Model..

[23] Livingston K.E., Stanczyk M.A., Burford N.T., Alt A., Canals M., Traynor J.R. (2018). Pharmacologic Evidence for a Putative Conserved Allosteric Site on Opioid Receptors. Mol. Pharmacol..

[24] Hothersall J.D., Torella R., Humphreys S., Hooley M., Brown A., McMurray G., Nickolls S.A. (2017). Residues W320 and Y328 within the binding site of the μ-opioid receptor influence opiate ligand bias. Neuropharmacology.

[25] Bock A., Merten N., Schrage R., Dallanoce C., Bätz J., Klöckner J., Schmitz J., Matera C., Simon K., Kebig A. (2012). The allosteric vestibule of a seven transmembrane helical receptor controls G-protein coupling. Nat. Commun..

[26] (2018). Schrödinger Release 2018-2: LigPrep, Schrödinger.

[27] (2018). Schrödinger Release 2018-2: Epik, Schrödinger.

[28] (2018). Schrödinger Release 2018-2: Glide, Schrödinger.

