# Supplementary material for: The Universal 3D QSAR Model for Dopamine D2 Receptor Antagonists
Source: Int J Mol Sci. 2019 Sep 14;20(18):4555. doi: 10.3390/ijms20184555 (PMC6770028; doi:10.3390/ijms20184555)
Supplement: Supplementary file 1 [file ijms-20-04555-s001.pdf]

Supplementary Information

The Universal 3D QSAR Model for Dopamine D2 Receptor Antagonists

Agata Zięba[1], Justyna Żuk[1], Damian Bartuzi[1], Dariusz Matosiuk[1], Antti Poso[2,3], and Agnieszka A. Kaczor [1,2,*]

[1] Department of Synthesis and Chemical Technology of Pharmaceutical Substances with Computer Modeling Laboratory, Faculty of Pharmacy with Division of Medical Analytics, 4A Chodzki St, PL-20059 Lublin, Poland; zieba.agata@gmail.com (A. Z.), j.siudem@gmail.com (J. Ż), damian.bartuzi@gmail.com (D.B.), dariusz.matosiuk@umlub.pl (D.M.).

[2] School of Pharmacy, University of Eastern Finland, Yliopistonranta 1, P.O. Box 1627, FI-70211 Kuopio, Finland; antti.poso@uef.fi (A.P.)

[3] University Hospital Tübingen Dept. of Internal Medicine VIII, Otfried-Müller-Strasse 14, 72076 Tübingen Germany; antti.poso@uef.fi (A.P.)

Table S1. The investigated compounds with experimental (exp.) and predicted (pred.) pIC_{50} values towards the dopamine D_2 receptor.

Compound number	Structure	pIC_{50} exp.	pIC_{50} pred.	Residual	Reference
	**Training set**				
**1**	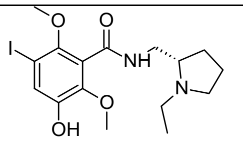	9.54	8.84	0.7	[1]
**2**	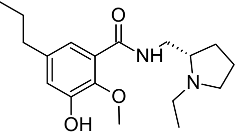	9.30	9.04	0.26	[1]
**3**	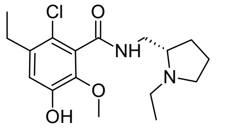	9.04	8.47	0.57	[1]
**4**	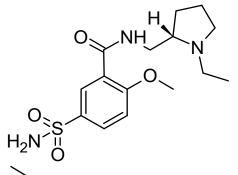	9.00	8.73	0.27	[1]
**6**	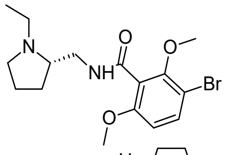	8.92	8.02	0.9	[1]
**8**	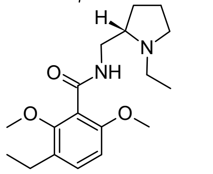	8.89	8.46	0.43	[1]

**9**	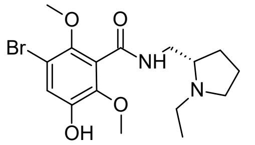	8.85	8.54	0.31	[1]
**10**	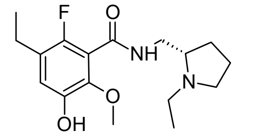	8.82	7.97	0.85	[1]
**11**	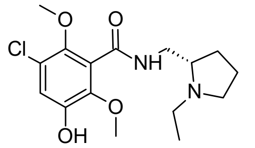	8.77	8.15	0.62	[1]
**12**	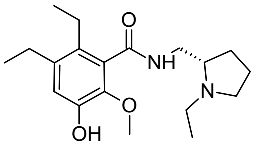	8.75	8.68	0.07	[1]
**13**	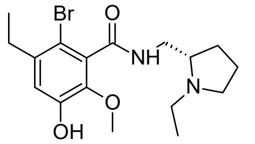	8.64	8.09	0.55	[1]
**14**	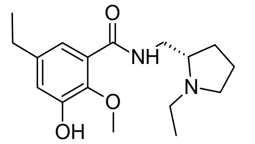	8.54	8.56	-0.02	[1]
**15**	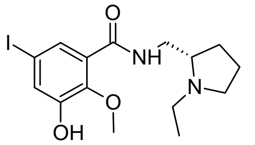	8.52	8.27	0.25	[1]
**16**	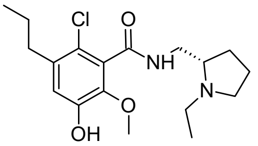	8.49	7.83	0.66	[1]
**17**	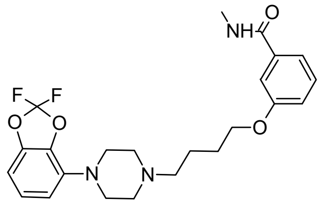	8.39	7.88	0.51	[2]
**18**	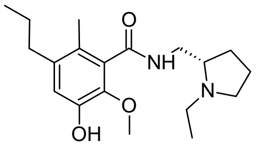	8.30	8.30	0.00	[1]
**19**	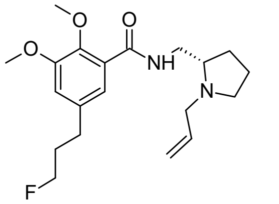	8.30	9.14	-0.84	[3]
**21**	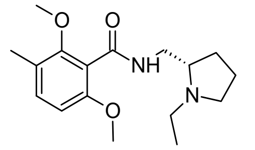	8.28	8.24	0.04	[1]

**22**	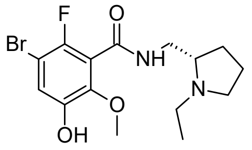	8.15	6.98	1.17	[1]
**23**	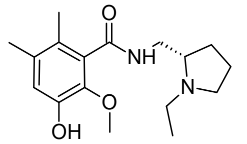	8.11	8.10	0.01	[1]
**24**	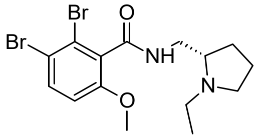	8.10	7.76	0.34	[1]
**25**	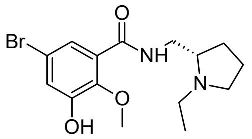	8.08	8.00	0.08	[1]
**26**	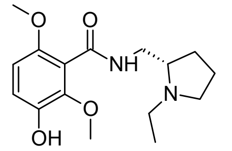	8.06	8.29	-0.23	[1]
**27**	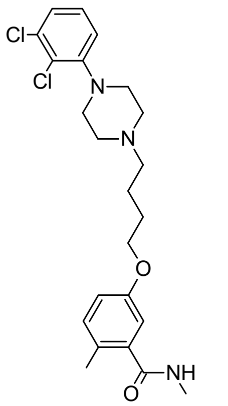	8.01	7.65	0.36	[2]
**28**	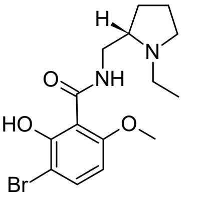	8.00	8.14	-0.14	[1]
**29**	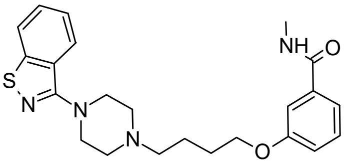	8.00	7.96	-0.04	[4]
**31**	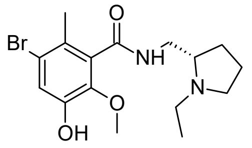	7.96	7.56	0.33	[1]
**32**	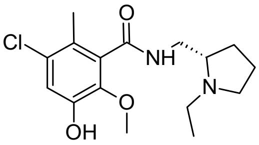	7.96	7.90	0.06	[1]

**33**	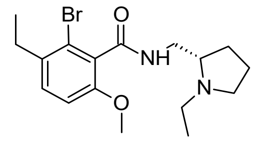	7.96	7.28	0.68	[1]
**34**	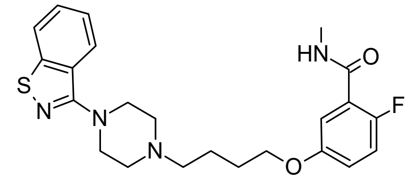	7.92	7.55	0.37	[4]
**35**	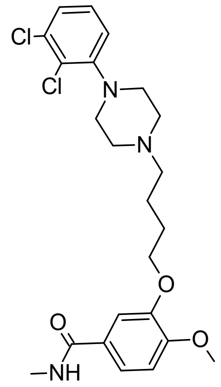	7.92	7.07	0.85	[2]
**36**	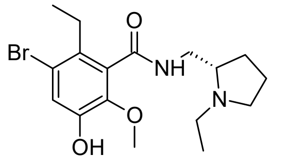	7.77	8.02	-0.25	[1]
**37**	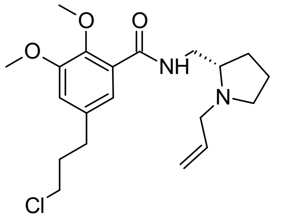	7.77	7.79	-0.02	[3]
**38**	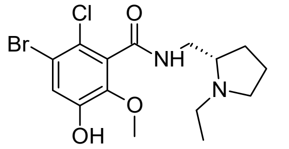	7.77	7.76	0.01	[1]
**39**	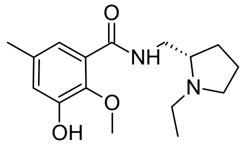	7.72	7.46	0.26	[1]
**40**	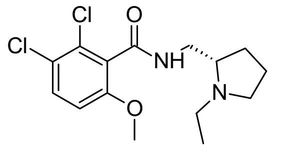	7.70	7.84	-0.14	[1]
**41**	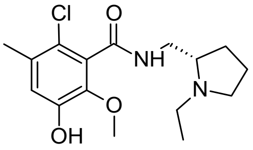	7.59	8.04	-0.45	[1]
**42**	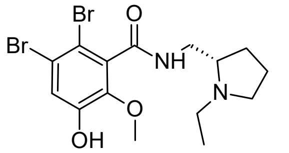	7.59	7.80	-0.21	[1]

**43**	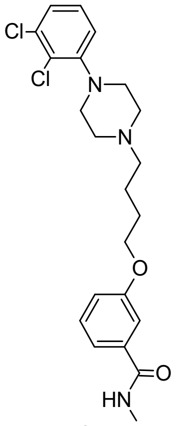	7.49	7.23	0.26	[2]
**44**	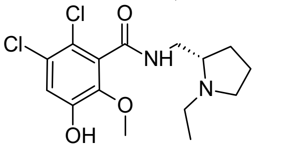	7.49	7.87	-0.38	[1]
**45**	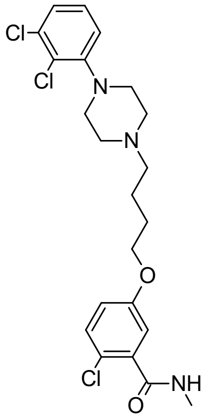	7.46	7.56	-0.1	[2]
**46**	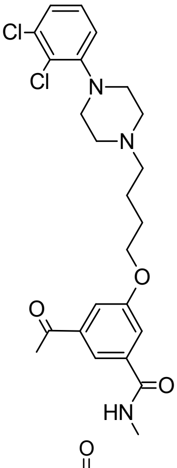	7.42	7.36	0.06	[2]
**47**	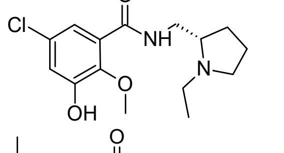	7.41	7.40	0.01	[1]
**48**	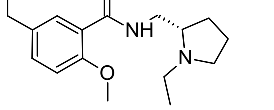	7.40	6.95	0.45	[1]

**49**	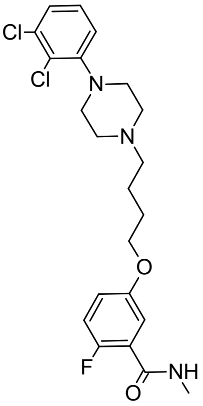	7.40	7.56	-0.16	[2]
**50**	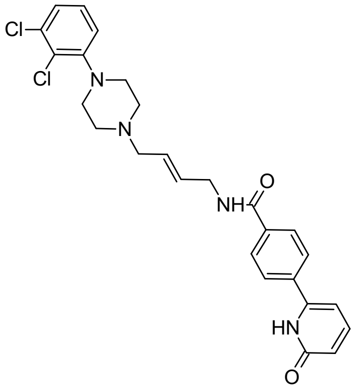	7.39	7.37	0.02	[5]
**51**	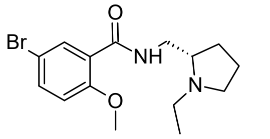	7.34	7.64	-0.3	[1]
**52**	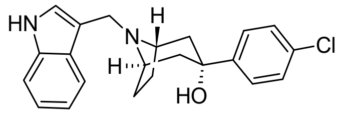	7.32	6.43	0.89	[6]
**53**	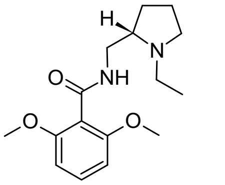	7.28	7.62	-0.34	[1]
**54**	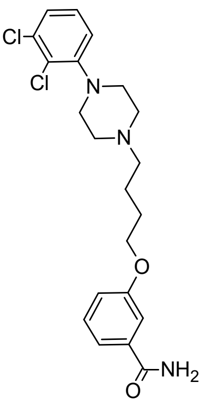	7.26	6.99	0.27	[2]
**55**	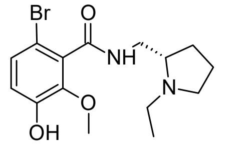	7.25	7.88	-0.63	[1]

**56**	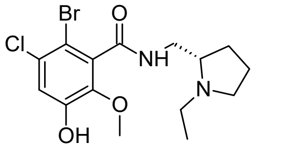	7.24	7.82	-0.58	[11]
**58**	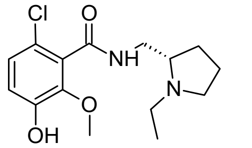	7.19	7.29	-0.1	[1]
**59**	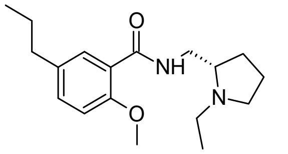	7.17	7.40	-0.23	[1]
**60**	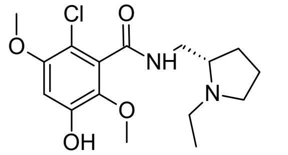	7.15	8.17	-1.02	[1]
**61**	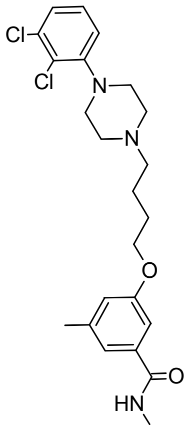	7.05	6.69	0.36	[2]
**62**	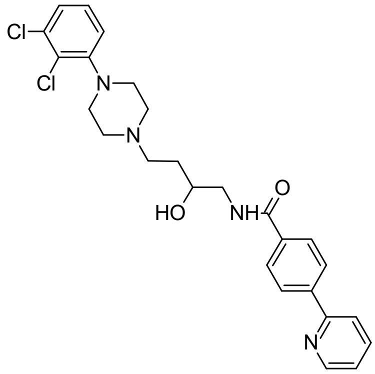	7.05	7.59	-0.44	[5]

**63**	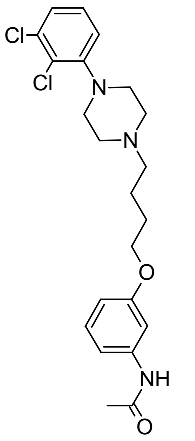	7.01	6.55	0.46	[2]
**64**	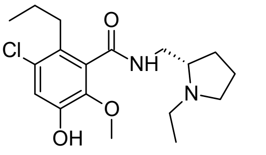	6.96	7.08	-0.12	[1]
**65**	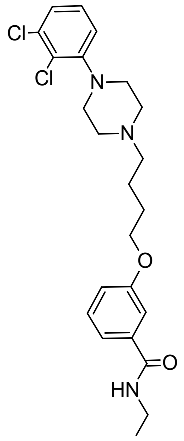	6.94	7.04	-0.10	[2]
**66**	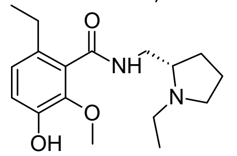	6.91	7.70	-0.79	[1]
**67**	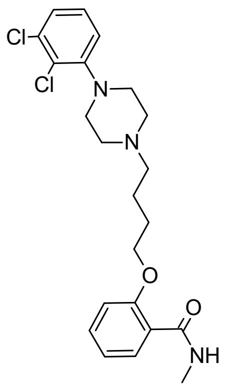	6.78	6.32	0.4	[2]

**68**	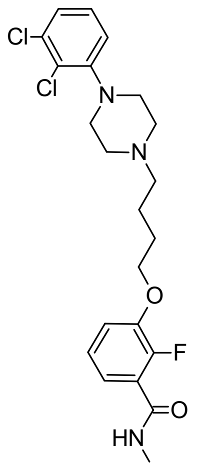	6.77	6.83	-0.06	[2]
**69**	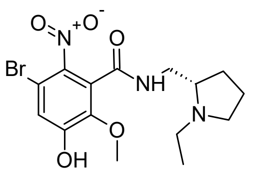	6.73	8.18	-1.45	[1]
**70**	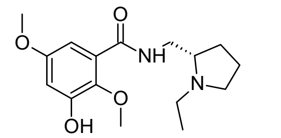	6.69	7.51	-0.82	[1]
**71**	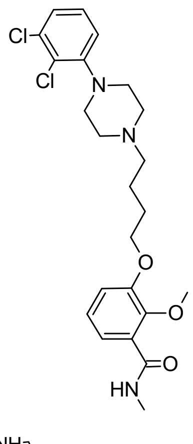	6.69	7.46	-0.77	[2]
**72**	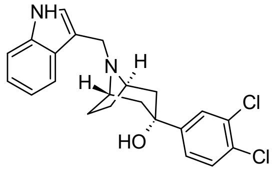	6.68	6.22	0.46	[6]

**73**	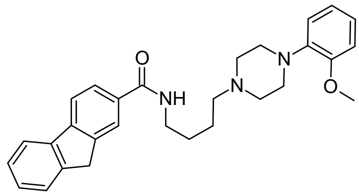	6.65	5.98	0.02	[16]
**74**	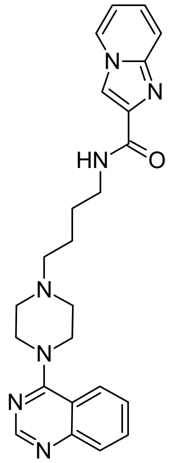	6.61	6.46	0.15	[7]
**75**	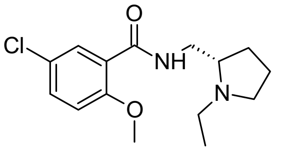	6.59	6.77	-0.18	[1]
**76**	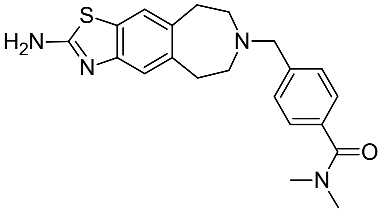	6.52	5.91	0.61	[8]
**77**	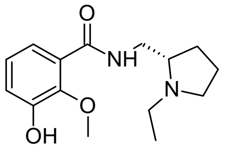	6.50	7.33	-0.83	[1]
**78**	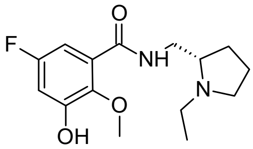	6.44	6.43	0.01	[1]
**79**	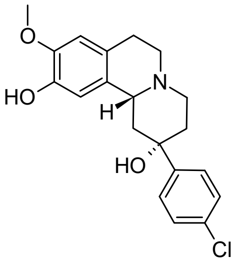	6.42	6.16	0.24	[9]
**80**	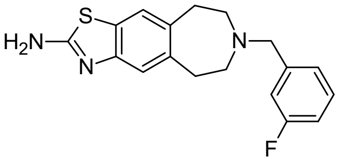	6.40	5.77	0.63	[8]

**81**	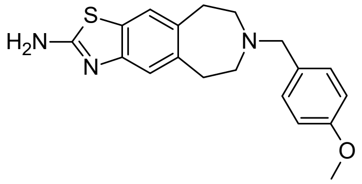	6.40	6.18	0.22	[8]
**82**	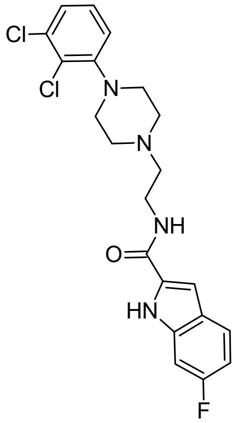	6.25	6.21	0.04	[10]
**83**	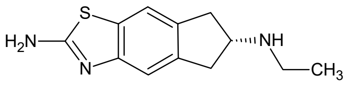	6.17	6.33	-0.16	[18]
**84**	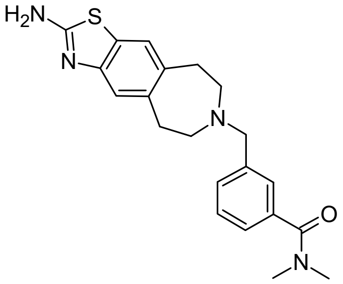	6.00	6.12	-0.12	[8]
**85**	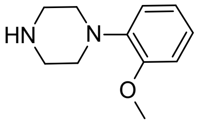	6.00	6.02	-0.02	[11]
**86**	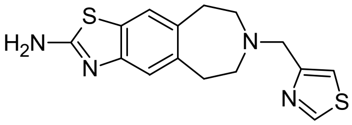	5.92	5.50	0.42	[8]
**87**	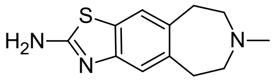	5.85	5.41	0.44	[8]
**88**	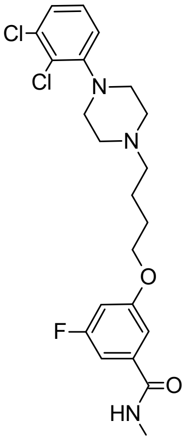	5.80	6.84	-1.04	[2]

**89**	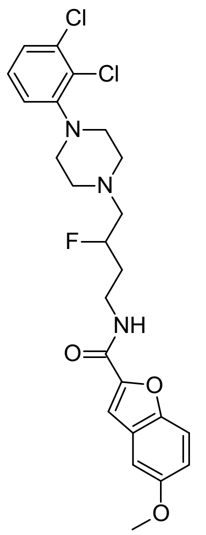	5.80	6.57	-0.77	[12]
**90**	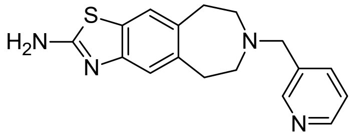	5.77	5.84	-0.11	[8]
**91**	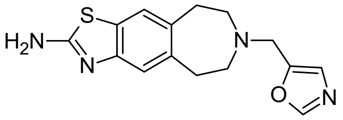	5.60	5.65	-0.05	[8]
**92**	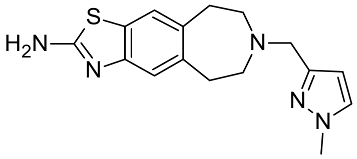	5.36	5.33	0.03	[8]
**93**	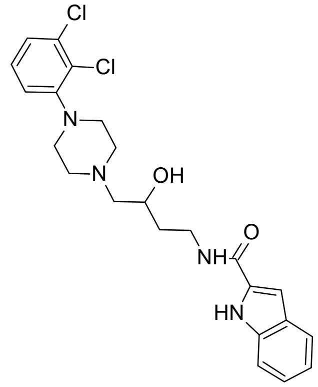	5.36	5.78	-0.42	[10]
**95**	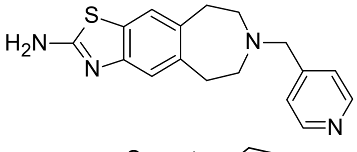	5.35	5.64	-0.29	[8]
**96**	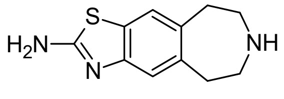	5.12	4.94	0.06	[8]
**97**	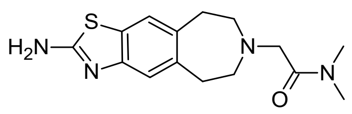	5.11	5.37	-0.37	[8]
**98**	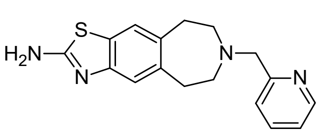	5.10	5.40	-0.30	[8]

**109**	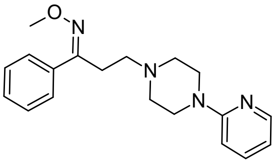	5.00	5.10	-0.10	[13]
**110**	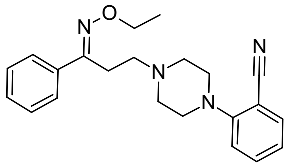	5.00	5.19	-0.19	[13]
**111**	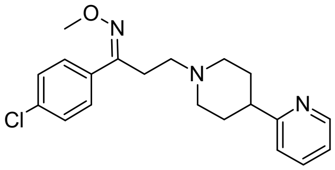	5.00	5.34	-0.34	[13]
**112**	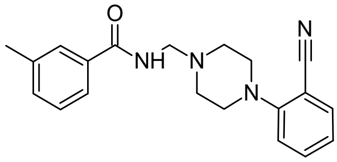	5.00	5.27	-0.27	[13]
**113**	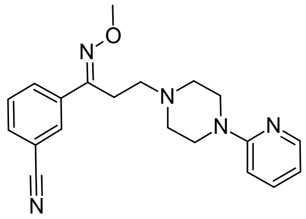	5.00	4.86	0.14	[13]
**114**	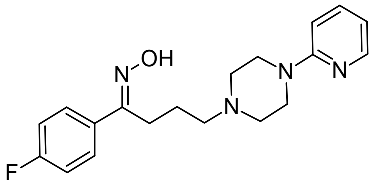	5.00	4.95	0.05	[13]
**115**	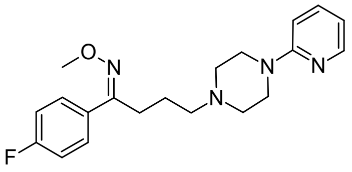	5.00	5.59	-0.59	[13]
**116**	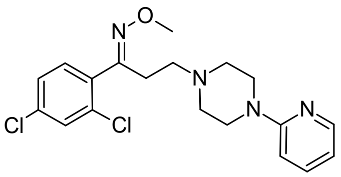	5.00	5.12	-0.12	[13]
**117**	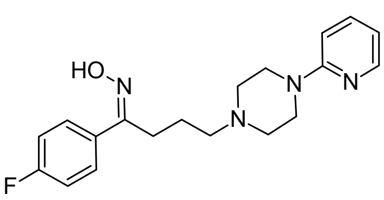	5.00	5.05	-0.05	[13]

**118**	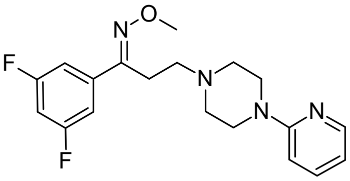	5.00	4.88	0.12	[13]
**119**	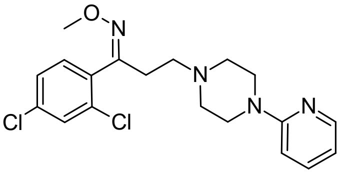	5.00	4.74	0.26	[13]
**120**	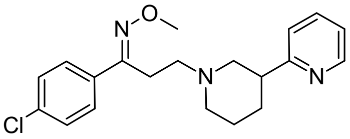	5.00	4.85	0.15	[13]
**121**	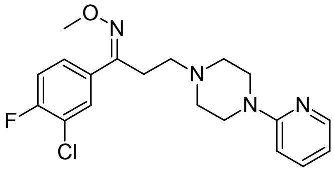	5.00	4.86	0.14	[13]
**122**	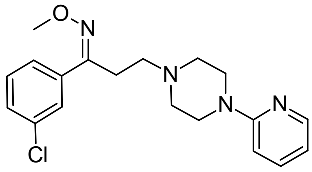	5.00	4.95	0.05	[13]
**123**	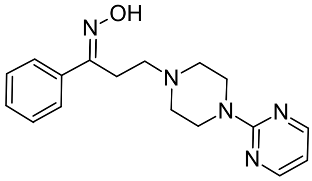	5.00	4.98	0.02	[13]
**124**	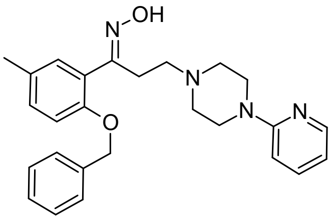	5.00	5.30	-0.30	[13]
**125**	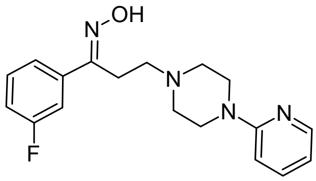	5.00	4.99	0.01	[13]
**126**	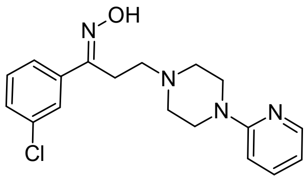	5.00	4.95	0.05	[13]
**127**	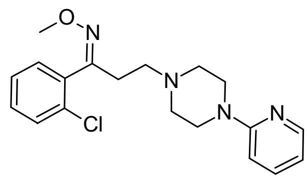	5.00	4.89	0.11	[13]

**128**	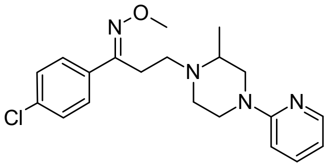	5.00	5.08	-0.08	[13]
**129**	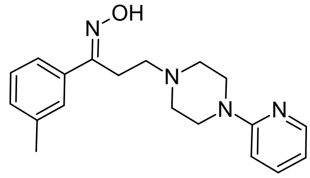	5.00	5.09	-0.09	[13]
**130**	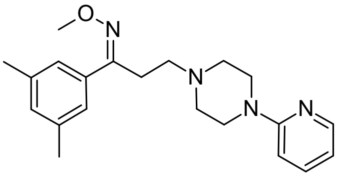	5.00	5.41	-0.41	[13]
**131**	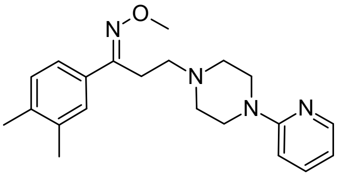	5.00	4.77	0.23	[13]
**132**	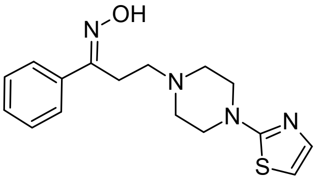	5.00	4.81	0.19	[13]
**133**	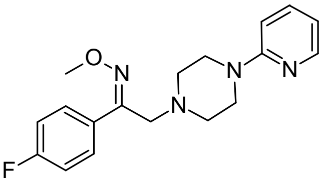	5.00	5.13	-0.13	[13]
**134**	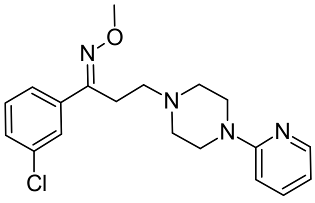	5.00	5.08	-0.08	[13]
**135**	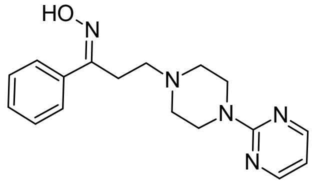	5.00	4.75	0.25	[13]
**136**	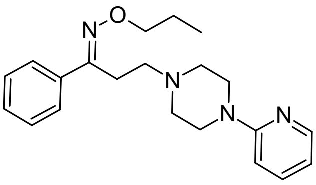	5.00	4.82	0.18	[13]

**137**	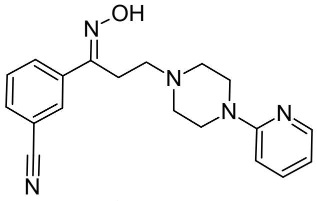	5.00	5.27	-0.27	[13]
**138**	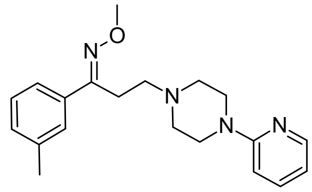	5.00	5.09	-0.09	[13]
**139**	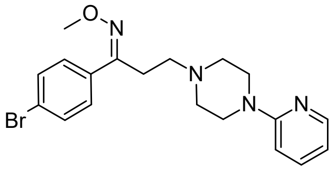	5.00	5.14	-0.14	[13]
**140**	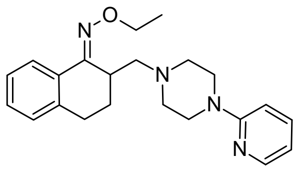	5.00	4.91	0.09	[13]
**141**	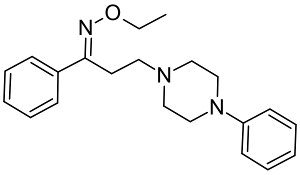	5.00	5.86	-0.86	[13]
**142**	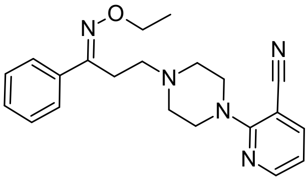	5.00	5.05	-0.05	[13]
**143**	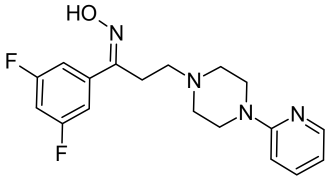	5.00	5.03	-0.03	[13]
**144**	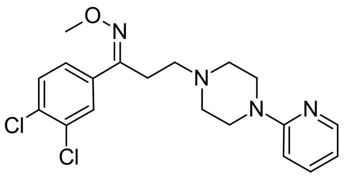	5.00	5.08	-0.08	[13]
**145**	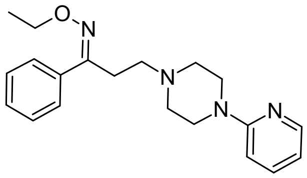	5.00	5.01	-0.01	[13]
**146**	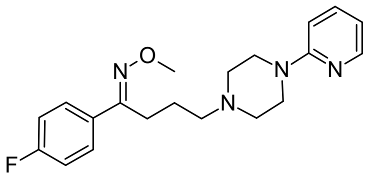	5.00	4.87	0.13	[13]

**147**	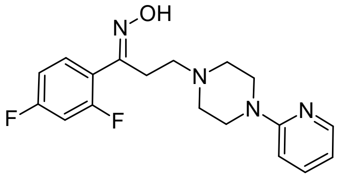	5.00	5.23	-0.23	[13]
**148**	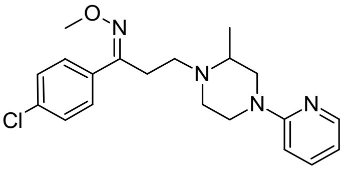	5.00	5.09	-0.09	[13]
**149**	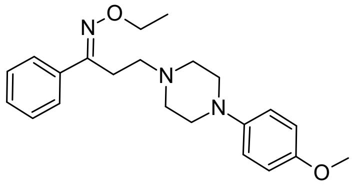	5.00	5.04	-0.04	[13]
**150**	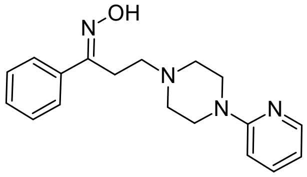	5.00	4.74	0.26	[13]
**151**	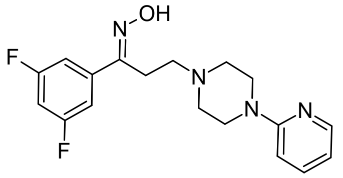	5.00	5.02	-0.02	[13]
**152**	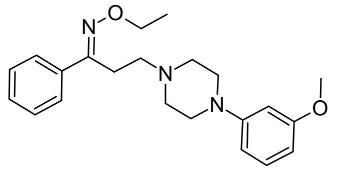	5.00	5.03	-0.03	[13]
**153**	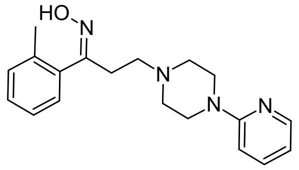	5.00	4.70	0.30	[13]
**154**	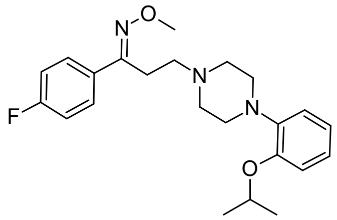	5.00	4.76	0.24	[13]
**155**	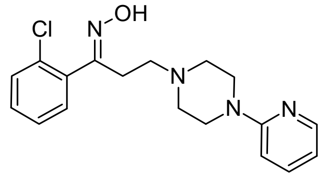	5.00	4.97	0.03	[13]
**156**	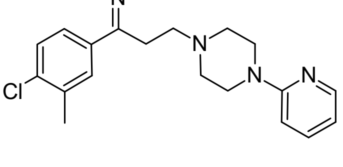	5.00	5.13	-0.13	[13]

**157**	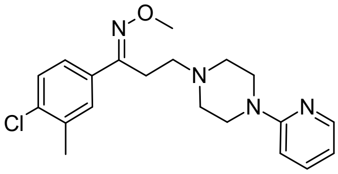	5.00	4.81	0.19	[13]
**158**	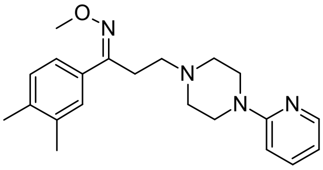	5.00	5.47	-0.47	[13]
**159**	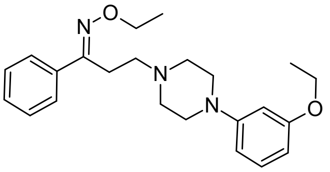	5.00	5.09	-0.09	[8]
**160**	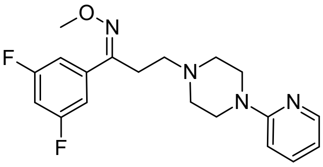	5.00	4.97	0.03	[13]
**161**	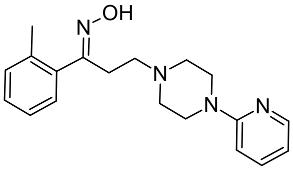	5.00	5.04	-0.04	[13]
**162**	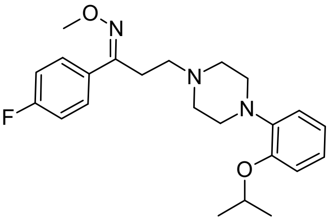	5.00	4.94	0.06	[8]
**163**	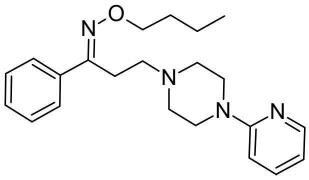	5.00	4.75	0.25	[13]
**164**	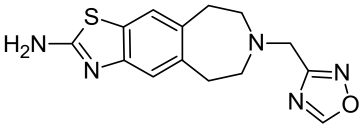	5.00	5.31	-0.31	[18]
**165**	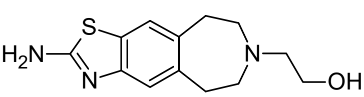	5.00	5.43	-0.43	[13]
**166**	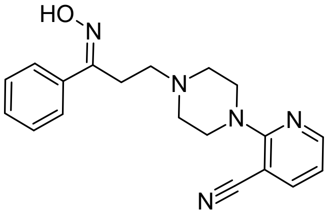	5.00	4.75	0.25	[13]

**167**	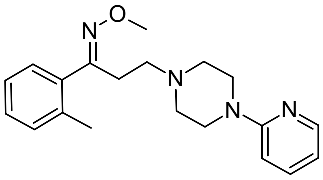	5.00	4.86	0.14	[13]
**168**	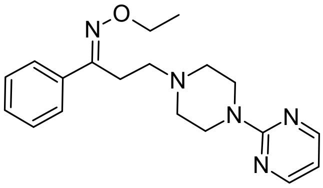	5.00	4.55	0.45	[13]
**169**	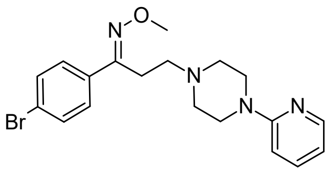	5.00	4.84	0.16	[13]
**170**	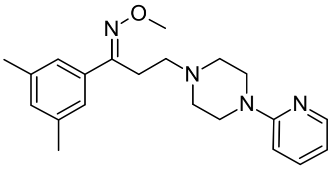	5.00	5.41	-0.41	[13]
**171**	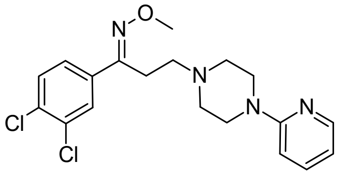	5.00	4.90	0.10	[13]
**172**	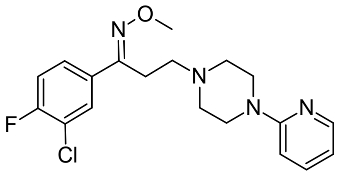	5.00	5.28	-0.28	[13]
**173**	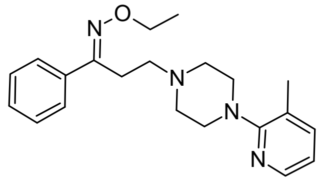	5.00	5.34	-0.34	[13]
**174**	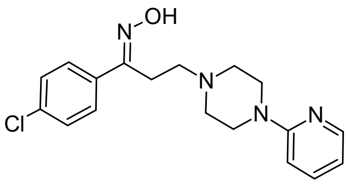	5.00	5.14	-0.14	[13]
**175**	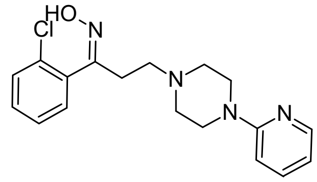	5.00	5.30	-0.30	[23]
**176**	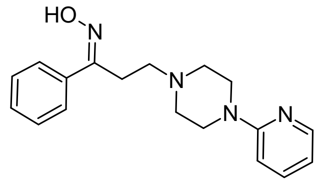	5.00	5.55	-0.55	[13]

		**Test set**			
**5**	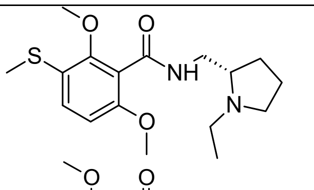	8.96	8.87	0.09	[1]
**7**	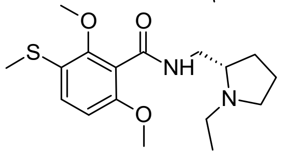	8.89	8.90	-0.01	[11]
**20**	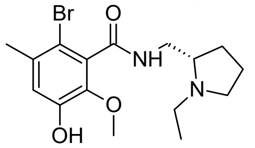	8.26	8.13	0.13	[1]
**30**	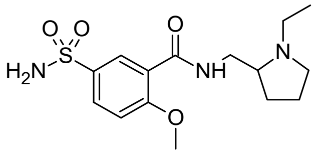	7.99	7.72	0.27	[14]
**57**	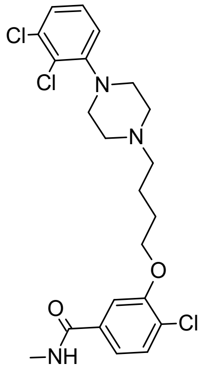	7.23	6.94	0.29	[2]
**94**	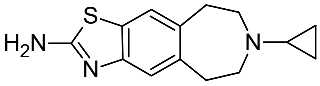	5.35	4.79	0.56	[3]
**99**	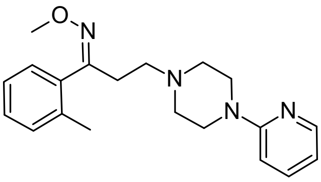	5.00	5.44	-0.44	[13]
**100**	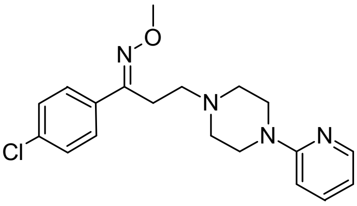	5.00	5.57	-0.57	[13]
**101**	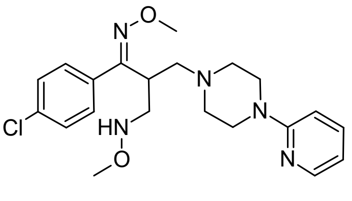	5.00	4.59	0.41	[13]

**102**	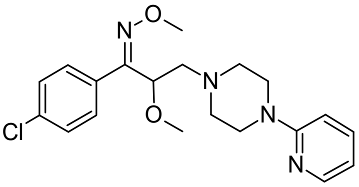	5.00	5.13	-0.13	[13]
**103**	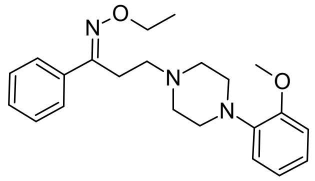	5.00	4.52	0.48	[13]
**104**	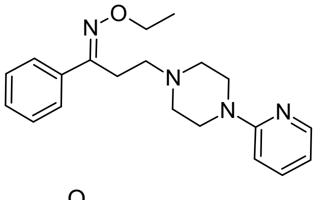	5.00	4.97	0.03	[13]
**105**	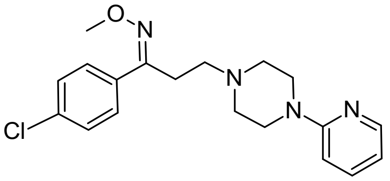	5.00	5.07	-0.07	[13]
**106**	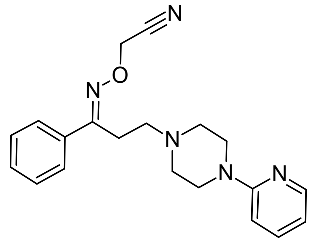	5.00	5.65	-0.65	[13]
**107**	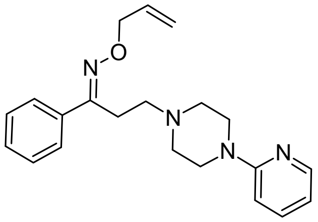	5.00	5.65	-0.65	[13]
**108**	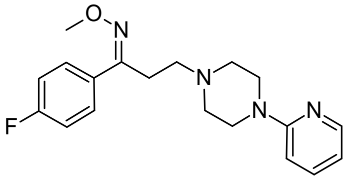	5.00	5.16	-0.16	[13]

References

1. Fatemi, M.H.; Dorostkar, F. QSAR prediction of D2 receptor antagonistic activity of 6-methoxy benzamides. *Eur. J. Med. Chem.* **2010**, *45*, 4856–4862.
2. Yang, F.; Jiang, X.; Li, J.; Wang, Y.; Liu, Y.; Bi, M.; Wu, C.; Zhao, Q.; Chen, W.; Yin, J.; et al. Synthesis, structure–activity relationships, and biological evaluation of a series of benzamides as potential multireceptor antipsychotics. *Bioorg. Med. Chem. Lett.* **2016**, *26*, 3141–3147.
3. Kim, K.; Miller, N.R.; Sulikowski, G.A.; Lindsley, C.W. A new multi-gram synthetic route to labeling precursors for the D2/3 PET agent 18F-fallypride. *Bioorg. Med. Chem. Lett.* **2008**, *18*, 4467–4469.
4. Grundt, P.; Carlson, E.E.; Cao, J.; Bennett, C.J.; McElveen, E.; Taylor, M.; Luedtke, R.R.; Newman, A.H. Novel Heterocyclic Trans Olefin Analogues of N-{4-[4-(2,3-Dichlorophenyl)piperazin-1-yl]butyl}arylcarboxamides as Selective Probes with High Affinity for the Dopamine D3 Receptor. *J. Med. Chem.* **2005**, *48*, 839–848.

5. Grundt, P.; Prevatt, K.M.; Cao, J.; Taylor, M.; Floresca, C.Z.; Choi, J.-K.; Jenkins, B.G.; Luedtke, R.R.; Newman, A.H. Heterocyclic Analogues of N-(4-(4-(2,3-Dichlorophenyl)piperazin-1-yl)butyl)arylcarboxamides with Functionalized Linking Chains as Novel Dopamine D3 Receptor Ligands: Potential Substance Abuse Therapeutic Agents. *J. Med. Chem.* **2007**, *50*, 4135–4146.
6. Paul, N.M.; Taylor, M.; Kumar, R.; Deschamps, J.R.; Luedtke, R.R.; Newman, A.H. Structure−Activity Relationships for a Novel Series of Dopamine D2-like Receptor Ligands Based on N-Substituted 3-Aryl-8-azabicyclo[3.2.1]octan-3-ol. *J. Med. Chem.* **2008**, *51*, 6095–6109.
7. Ananthan, S.; Saini, S.K.; Zhou, G.; Hobrath, J.V.; Padmalayam, I.; Zhai, L.; Bostwick, J.R.; Antonio, T.; Reith, M.E.A.; McDowell, S.; et al. Design, Synthesis, and Structure–Activity Relationship Studies of a Series of [4-(4-Carboxamidobutyl)]-1-arylpiperazines: Insights into Structural Features Contributing to Dopamine D3 versus D2 Receptor Subtype Selectivity. *J. Med. Chem.* **2014**, *57*, 7042–7060.
8. Urbanek, R.A.; Xiong, H.; Wu, Y.; Blackwell, W.; Steelman, G.; Rosamond, J.; Wesolowski, S.S.; Campbell, J.B.; Zhang, M.; Brockel, B.; et al. Synthesis and SAR of aminothiazole fused benzazepines as selective dopamine D2 partial agonists. *Bioorg. Med. Chem. Lett.* **2013**, *23*, 543–547.
9. Giovanni, A.; Roehr, J.; Dwyer, S.; Neuenschwander, K.; Scotese, A.; Moorcroft, N.D.; Davis, L.; Gao, Z. Design and synthesis of D1 agonist/D2 antagonist for treatment of schizophrenia. *Bioorg. Med. Chem. Lett.* **2013**, *23*, 1498–1501.
10. Boateng, C.A.; Bakare, O.M.; Zhan, J.; Banala, A.K.; Burzynski, C.; Pommier, E.; Keck, T.M.; Donthamsetti, P.; Javitch, J.A.; Rais, R.; et al. High Affinity Dopamine D3 Receptor (D3R)-Selective Antagonists Attenuate Heroin Self-Administration in Wild-Type but not D3R Knockout Mice. *J. Med. Chem.* **2015**, *58*, 6195–6213.
11. Szabo, M.; Klein Herenbrink, C.; Christopoulos, A.; Lane, J.R.; Capuano, B. Structure–Activity Relationships of Privileged Structures Lead to the Discovery of Novel Biased Ligands at the Dopamine D2 Receptor. *J. Med. Chem.* **2014**, *57*, 4924–4939.
12. Banala, A.K.; Levy, B.A.; Khatri, S.S.; Furman, C.A.; Roof, R.A.; Mishra, Y.; Griffin, S.A.; Sibley, D.R.; Luedtke, R.R.; Newman, A.H. N-(3-fluoro-4-(4-(2-methoxy or 2,3-dichlorophenyl)piperazine-1-yl)butyl)arylcarboxamides as selective dopamine D3 receptor ligands: critical role of the carboxamide linker for D3 receptor selectivity. *J. Med. Chem.* **2011**, *54*, 3581–3594.
13. Kolasa, T.; Matulenko, M.A.; Hakeem, A.A.; Patel, M.V.; Mortell, K.; Bhatia, P.; Henry, R.; Nakane, M.; Hsieh, G.C.; Terranova, M.A.; et al. 1-Aryl-3-(4-pyridine-2-ylpiperazin-1-yl)propan-1-one Oximes as Potent Dopamine D4 Receptor Agonists for the Treatment of Erectile Dysfunction. *J. Med. Chem.* **2006**, *49*, 5093–5109.
14. Vullo, D.; Innocenti, A.; Nishimori, I.; Pastorek, J.; Scozzafava, A.; Pastoreková, S.; Supuran, C.T. Carbonic anhydrase inhibitors. Inhibition of the transmembrane isozyme XII with sulfonamides-a new target for the design of antitumor and antiglaucoma drugs? *Bioorg. Med. Chem. Lett.* **2005**, *15*, 963–969.
